# Case report: A case of primary renal osteosarcoma

**DOI:** 10.1097/MD.0000000000039024

**Published:** 2024-07-26

**Authors:** Wei Su, Hao Fu, Xiangming Mao

**Affiliations:** a Department of Urology, Affiliated Nanhua Hospital, University of South China, Hengyang, China; b Department of Urology, Zhujiang Hospital, Southern Medical University, Guangzhou, China.

**Keywords:** chemotherapy, nephrectomy, primary renal osteosarcoma, targeted therapy

## Abstract

**Rationale:**

Primary renal osteosarcoma is an exceedingly rare malignant tumor. Fewer than 30 cases have been reported in the literature since 1936. Furthermore, it has a high risk of metastasis and a poor overall prognosis rate.

**Patient’s concerns:**

In this report, we present a case of osteosarcoma originating from the left kidney (21 cm × 18 cm × 11 cm) with adhesions of the descending colon mesentery and the abdominal wall. A 63-year-old male patient presented with flank pain and gross hematuria.

**Diagnoses:**

A computed tomography scan revealed that the lesion with irregular margins was observed at the lower pole of the left kidney. Enhanced CT scan showed significant inhomogeneous enhancement of the lesion, with non-enhancing necrotic areas. A radical nephrectomy was performed. The immunohistochemistry results support the diagnosis of osteosarcoma.

**Interventions:**

Postoperatively, the patient participated in clinical trials and received treatment with a PD-1 antibody (2 weeks/once).

**Outcomes:**

At the one-year follow-up, the patient reported late-stage systemic cancer-related pain, necessitating daily pain relief medication. Unfortunately, the patient passed away 18 months after the surgery.

**Lessons:**

The case reported here is an exceedingly rare malignant osteosarcoma that originated from the kidney with the invasion of the descending colon mesentery and the abdominal wall. The patient received treatment with a PD-1 antibody (2 weeks/once). However, the patient passed away 18 months after the surgery. With the application of genetic testing technology and advancements in molecular biology, it was expected that specific targeted therapies for this condition would emerge in the future.

## 1. Introduction

Primary renal osteosarcoma presents as an exceedingly rare malignant tumor with nonspecific clinical manifestations. Fewer than 30 cases have been reported so far.^[1,2]^ It accounts for less than 4% of osteosarcomas and less than 1% of soft tissue sarcomas.^[3]^ The condition can manifest in individuals aged 29 to 82 years.^[1]^ The etiology and pathogenesis remain unclear. The “metaplastic theory” is often cited,^[4,5]^ suggesting that connective tissue can transform into embryonic mesenchyme in a specific microenvironment, possessing the ability to differentiate into osteoblasts and form bone tissue. Patients are usually diagnosed at an advanced stage and 32% of patients have metastases_,_^[1]^ so the overall prognosis rate is very poor. In this article, we present a case of primary osteosarcoma of the kidney and review its clinical features, diagnosis, and treatment options.

## 2. Case presentation

**Figure 1.**
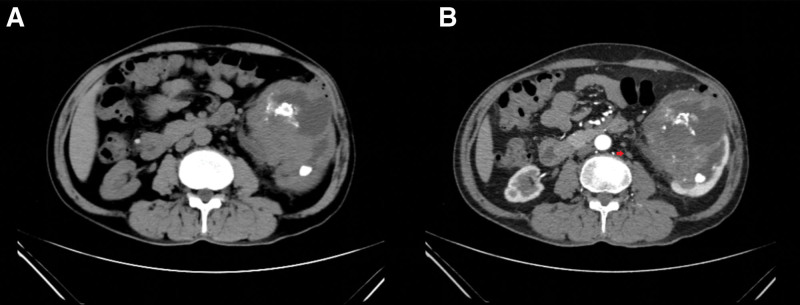
(A) On CT plain scan, an irregular, ill-defined mass measuring 8.8 cm × 8.2 cm is observed at the lower pole of the left kidney, with multiple scattered calcifications. (B) Contrast-enhanced scan shows marked heterogeneous enhancement of the mass, with areas of non-enhancing necrosis. The red arrow indicates an enlarged retroperitoneal lymph node.

The patient, a 63-year-old male, was admitted on September 25, 2019, reporting “left-sided flank pain accompanied by gross hematuria for six months, worsening over the last three days.” He had a history of left pyelolithotomy for stone removal. Physical examination revealed no specific findings. Laboratory Tests: Carcinoembryonic antigen (CEA) was 5.3μg/L (Reference Range: 0-5μg/L), free prostate-specific antigen (fPSA) was 0.58μg/L (Reference Range: 0-0.93μg/L), total prostate-specific antigen (tPSA) was 2.84μg/L (Reference Range: 0-4μg/L) and fPSA to tPSA ratio was 20.4% (Reference Range > 25%). Routine blood test: Hemoglobin was 148g/L, white blood cells were 6.5 × 10^9^/L and Platelets were 323 × 10^9^/L. Creatinine (Cr) was 113.9umol/L (Reference Range: 70-115 µmol/L). Postoperative Cr increased to 145umol/L. Abdominal Plain X-ray: The left kidney appeared enlarged with multiple irregular high-density lesions, and the borders were unclear. CT Scan: A lesion measuring approximately 88mm × 82mm × 82mm with irregular margins was observed at the lower pole of the left kidney, with an unclear border and a CT value of approximately 38 Hounsfield Units (Hu). The density within the lesion was non-uniform, with small areas of slightly lower density and multiple scattered calcifications (Fig. 1A). Enhanced CT scan revealed significant inhomogeneous enhancement of the lesion, with non-enhancing necrotic areas (Fig. 1B). The upper segment of the left ureter showed thickening and a rough surface, along with moderate to severe dilation and fluid accumulation in the left renal pelvis and calyces. Multiple nodular and dense shadows were seen in the lower pole of the left kidney. Retroperitoneal lymph nodes were enlarged (Fig. 1B). Chest CT: Multiple nodular shadows were observed in the lower lobes of both lungs with clear borders. Thickening of the left lower pleura was noted. CT angiography: The vascular supply to the left renal mass originated from branches of the left renal artery. Renal Dynamic Imaging: The left renal mass was evident. Renal blood flow and perfusion appeared generally normal. Glomerular filtration rates (GFR) for the left and right kidneys were 47.08 mL/min and 30.78 mL/min, respectively.

Clinical Diagnosis: Left Renal Tumor. The patient underwent radical left nephrectomy on September 29, 2019. Laparoscopic exploration during the surgery revealed significant adhesions between the descending colon mesentery and the abdominal wall. Due to the tumor’s size and severe adhesions to surrounding intestines, laparoscopic procedures were limited, and an open surgical approach was adopted. Upon exposure of the tumor, it was found to be severely adherent to the renal pedicle and descending colon. Intraoperative Frozen Pathology: Left Kidney: Low-grade carcinoma. Descending Colon Mesentery: Inflammatory necrotic tissue.

**Figure 2.**
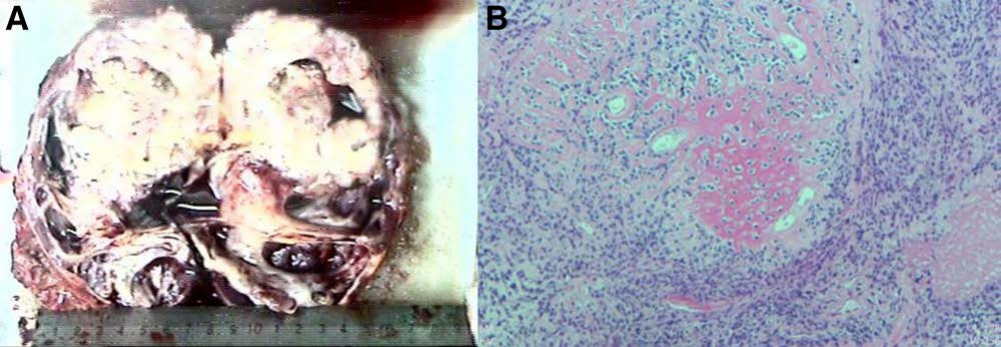
(A) Gross examination reveals a gray-white to gray-yellow appearance of the mass, with local areas showing evidence of hemorrhage. (B) Microscopically, the tumor tissue exhibits nest-like or patchy distribution, with the presence of red-stained bone-like tissue between tumor cells (Hematoxylin and Eosin staining, magnification ×100).

Postoperatively, the patient experienced a smooth recovery and was discharged on the 6th day after surgery. The routine pathological report described the tumor as measuring approximately 9.0 × 7.0 × 7.0 cm grossly, with a gray-white to gray-yellow cut surface and areas of hemorrhage (Fig. 2A). The descending colon mesentery exhibited gray-red tissue. Microscopically, the tumor tissue in the left kidney displayed nests and sheet-like arrangements, with some tumor cells exhibiting osteoid or red-stained bone-like, cartilage-like, or mucinous-like matrices (Fig. 2B). Few osteoclast-like giant cells were observed. The tissue in the descending colon mesentery showed a small amount of bone-like tissue, with scattered atypical cells. Immunohistochemistry results indicated positivity for Vim, CD99, SYN, and P53, while being negative for CK, CK7, CK20, CK5/6, and P63. The Ki-67 index was greater than 90%. The pathological diagnosis was left renal small-cell osteosarcoma with the descending colon mesentery invasion. Postoperatively, the patient received treatment with a PD-1 antibody (2 weeks/once). At the one-year follow-up, the patient reported late-stage systemic cancer-related pain, necessitating daily pain relief medication. Unfortunately, the patient passed away 18 months after the surgery.

## 3. Discussion and conclusions

In this case, the patient presented with involvement of the descending colon mesentery and lung metastases at the initial presentation, indicating a high degree of malignancy in the tumor. Some studies report that 32% of patients have distant metastases at symptom onset, and despite aggressive treatment, 86% of patients experience local recurrence or distant metastasis.^[1]^

Elevated serum alkaline phosphatase levels have been suggested by Virgilio and others as a tool for diagnosing and monitoring recurrence or metastasis during follow-up in this disease.^[6]^ However, in the presented case, the patient’s alkaline phosphatase was within normal limits at the time of diagnosis. Ah-Chong and others reported cases with multiple bone metastases but normal serum alkaline phosphatase levels,^[7]^ indicating ongoing debate regarding the sensitivity and specificity of using serum alkaline phosphatase as a diagnostic and follow-up marker for this disease. Nevertheless, alkaline phosphatase levels can help assess the prognosis of extraosseous osteosarcoma.^[8]^ Primary renal osteosarcoma may present on CT as a soft tissue mass with variable density, irregular borders, internal areas of cotton-wool-like bone density, cystic changes, necrosis, and hemorrhage.^[9]^ The lesion often exhibits relatively lower density compared to adjacent muscle tissue. The possibility of this disease should be considered when the presence of irregularly distributed spots or patches of calcifications occurs in renal soft tissue masses. Under a microscope, the tumor can consist of various tumor cell shapes, including small round cells, spindle cells, osteoclast-like cells, epithelioid cells, or multinucleated cells.^[10,11]^ Typically, red-stained bone-like or cartilage-like matrix can be observed between tumor cells, with a significant amount of tumor bone present. Similar pathological changes are often found in most metastatic lesions. Since primary renal osteosarcoma originates from the stroma, positive staining for Vim in immunohistochemistry, along with a Ki-67 index exceeding 70%, and negative epithelial cell markers, as well as other cell molecular markers such as neuron-specific enolase, synaptophysin (SYN), and smooth muscle actin, can aid in diagnosis.

In the immunohistochemical analysis in this case, SYN demonstrated diffuse positivity. Differential diagnosis was essential, particularly in distinguishing it from primary renal small-cell neuroendocrine tumors. The primary method of differentiation relied on pathological examination. Small-cell neuroendocrine tumors typically presented under the microscope as tumor cells arranged in sheets or nests with a diffuse infiltrative pattern. Additionally, multifocal necrotic areas were often observed. The tumor cells exhibited small volume, resembling oat cells, with scant cytoplasm and nearly naked nuclei. They displayed deep nuclear staining, inconspicuous chromatin and nucleoli, and a high frequency of apoptotic and mitotic figures, including atypical mitoses.^[12]^ It’s worth noting that in the context of these tumor cells, osseous, cartilaginous stromal elements, or tumor bone were not observed. In addition to distinguishing them from neuroendocrine tumors, differentiation must also consider common renal clear cell carcinoma and secondary renal osteosarcoma. In renal clear cell carcinoma, ossification was exceedingly rare, and its immunohistochemical profile markedly differed from primary osteosarcomas due to its epithelial origin.^[1]^ Furthermore, lesions of secondary renal osteosarcoma were typically bilateral, multifocal, and often clinically asymptomatic. Radiologically, they may have exhibited extensive areas of calcification and occasionally presented a “sunburst” appearance on imaging, which could even be observable on plain radiographs.^[13]^

Due to the limited number of reported cases in the literature, a unified treatment protocol for this condition has not yet been established. Surgical resection of the primary lesion remained the primary treatment approach. Typically, when this condition was discovered, the tumor might have been large or locally adherent, necessitating potential extension of the surgical incision or cystic fluid aspiration for decompression to achieve adequate exposure of the surgical field.^[5]^ Early diagnosis of the tumor and prompt surgical intervention could significantly improve patient prognosis.^[10]^ Compared to chemotherapy regimens for soft tissue sarcomas, platinum-based combination chemotherapy regimens might be more favorable for improving patient outcomes in the case of osteosarcomas.^[14]^ Unlike earlier cases where chemotherapy drugs were used as monotherapy for tumor treatment, recent studies have shown that a combination of various chemotherapy drugs and the targeted therapy Anlotinib could lead to a significant extension in disease-free survival for patients.^[1]^ This might be related to the choice of chemotherapy regimen and the application of targeted therapies. Although the effectiveness of targeted therapies for primary renal osteosarcoma still required further research, due to the aggressive nature of this condition, it was recommended to consider genetic testing of postoperative pathological samples and the selection of appropriate targeted therapies to improve disease-free and overall survival rates. In the present case, the patient received continued treatment with PD-1 monotherapy at our hospital. PD-1, as an immunotherapy approach for cancer treatment, had shown some therapeutic effect in the disease progression of osteosarcoma in recent clinical trials. However, it still lacked comprehensive clinical data support.^[15,16]^

Primary renal osteosarcoma tends to invade the perirenal fat, leading to local and distant metastasis, with a very high malignancy rate. It was often diagnosed in the late stages, and the most common sites for metastasis were the lungs, liver, colon, and ipsilateral adrenal gland.^[1,17]^ A study of 26 primary renal osteosarcoma patients reported that 22 of them died within six months, indicating a generally poor prognosis.^[5]^ Therefore, long-term follow-up observation was essential even after surgical removal of the tumor. With the application of genetic testing technology and advancements in molecular biology, it was expected that specific targeted therapies for this condition would emerge in the future.

## Acknowledgments

We would like to express our gratitude to the patient for granting permission to use their clinical data in this paper and for the publication of this research.

## Author contributions

**Data curation:** Wei Su, Hao Fu, Xiangming Mao.

**Formal analysis:** Wei Su, Hao Fu.

**Writing – original draft:** Wei Su, Xiangming Mao.
